# NPAS2 and PER2 are linked to risk factors of the metabolic syndrome

**DOI:** 10.1186/1740-3391-7-5

**Published:** 2009-05-26

**Authors:** Ani Englund, Leena Kovanen, Sirkku T Saarikoski, Jari Haukka, Antti Reunanen, Arpo Aromaa, Jouko Lönnqvist, Timo Partonen

**Affiliations:** 1 Department of Mental Health and Alcohol Research, National Public Health Institute, Mannerheimintie 166, FI-00300 Helsinki, Finland; 2 Department of Health and Functional Capacity National Public Health Institute, Mannerheimintie 166, FI-00300 Helsinki, Finland; 3 Department of Biostatistics and Epidemiology Cluster, International Agency for Research on Cancer, Lyon, France (Haukka)

## Abstract

**Background:**

Mammalian circadian clocks control multiple physiological events. The principal circadian clock generates seasonal variations in behavior as well. Seasonality elevates the risk for metabolic syndrome, and evidence suggests that disruption of the clockwork can lead to alterations in metabolism. Our aim was to analyze whether circadian clock polymorphisms contribute to seasonal variations in behavior and to the metabolic syndrome.

**Methods:**

We genotyped 39 single-nucleotide polymorphisms (SNP) from 19 genes which were either canonical circadian clock genes or genes related to the circadian clockwork from 517 individuals drawn from a nationwide population-based sample. Associations between these SNPs and seasonality, metabolic syndrome and its risk factors were analyzed using regression analysis. The p-values were corrected for multiple testing.

**Results:**

Our findings link circadian gene variants to the risk factors of the metabolic syndrome, since *Npas2 *was associated with hypertension (P-value corrected for multiple testing = 0.0024) and *Per2 *was associated with high fasting blood glucose (P-value corrected for multiple testing = 0.049).

**Conclusion:**

Our findings support the view that relevant relationships between circadian clocks and the metabolic syndrome in humans exist.

## Background

Circadian clocks regulate the timing of biological events including the sleep-wake cycle, energy metabolism, and secretion of hormones. The principal clock conducting the circadian system is located in the suprachiasmatic nuclei of the anterior hypothalamus. From the brain, information is sent out to regulate and reset the peripheral clocks [1]. Seasonal variations in behavior are generated by the principal clock as well [2]. Light exposures stimulate the principal clock through pathways from the retina, and the most important cues for reset of the principal circadian clock are the light-dark transitions, while the peripheral clocks are set by metabolic signals in response to feeding cycles [3]. With shortage of daylight, the metabolic cycles may take over and serve as the standard for the circadian clockwork [4]. In hibernating mammals, the metabolic futile cycle can provide the animal with those circadian signals needed for reset [5]. When there exists no light-dark transitions to reset the principal clock, reindeer living above the Arctic Circle use the metabolic cycles as the reference instead [6].

The molecular circadian clock consists of multiple positive and negative feedback loops that generate the 24-hour oscillation of target genes. In the positive loop NPAS2 (MOP4) protein [7], which plays an overlapping role with the CLOCK protein [8], pairs up with ARNTL (BMAL1 or MOP3) protein. These heterodimers activate the transcription of target genes (for review, see [9]). Downstream, PER and CRY proteins pair up and execute the negative loop. Nuclear receptor co-activators and repressors and several post-transcriptional modifications are necessary for clock precision. In addition, clockwork output molecules can provide an input to the following cycles [10].

Circadian clocks and energy metabolism are linked because the disruptions of the clockwork lead to alterations in metabolism and vice versa (for review, see [11]). Mutation in the *Clock *gene leads to metabolic syndrome in mice [12], and in humans *Clock *polymorphisms have been associated with obesity and metabolic syndrome [13,14]. Cellular metabolic states can serve as a link between stimuli from the habitat and drive for the clockwork, because the reduced forms of nicotinamide adenine dinucleotide cofactors stimulate DNA binding of the NPAS2-ARNTL [15] and CLOCK-ARNTL [16] heterodimers, whereas the oxidized forms inhibit the binding [17]. *Npas2*-deficient mice have reduced ability to adapt to restricted feeding [18], whereas *Clock*-deficient mice adapt to it even better than do wild-type mice [19], suggesting a key role of NPAS2.

Herein, we hypothesized that circadian clock polymorphisms contribute to the routine seasonal variations and to the metabolic syndrome. Our earlier finding that seasonality was associated with the metabolic syndrome [20], gave a rationale for the current study.

## Methods

This study was part of a nationwide health interview and examination survey, the Health 2000 Study, which was carried out in Finland, a north-eastern (60–70°N, 20–31°E) European country with about 5 million inhabitants, from September 2000 to June 2001. The two-stage stratified cluster sampling design was planned by Statistics Finland. The sampling frame comprised adults living in mainland Finland. This frame was regionally stratified according to the five university hospital regions, or catchments areas, each containing roughly one million inhabitants. From each university hospital region, 16 health care districts were sampled as clusters (80 health care districts in the whole country, including 160 municipalities, or groups of municipalities with joint primary care). The 15 biggest health care districts in the country were all selected in the sample and their sample sizes were proportional to population size. The remaining 65 health care districts were selected by systematic probability proportional to size sampling in each stratum, and their sample sizes (ranging from 50 to 100) were equal within each university hospital region, the total number of persons drawn from a university hospital region being proportional to the corresponding population size. The 80 health care districts were the primary sampling units, and the ultimate sampling units were persons who were selected by systematic sampling from the health centre districts. From these 80 health care districts, a random sample of individuals was drawn using the data provided by Population Register Centre. Its population information system contains the official information for the whole country on the Finnish citizens and aliens residing permanently in Finland. All the persons aged 30 and over (n = 8028) who were identified from the nationally representative sample by The Social Insurance Institution of Finland were contacted in person. Interviewers attended training sessions on the specific themes that were to be covered in the computer-assisted interviews. Of the final sample of 7979 persons, 6986 (88%) were interviewed at home or institution face to face and 6354 (80%) attended the health status examination in a local health center or equal setting, while 416 took part in the health status examination at home or in an institution. Overall, 84% participated either in the health status examination proper or in the examination at home. All the methods are reported in more detail on the Internet site of the Health 2000 .

### Phenotype data

All participants had been asked to come to the health status examination fasting at least 4 hours and without drinking on the same day. In the laboratory, a nurse recorded how these instructions had been followed and then took the blood samples. The samples were centrifuged at the examination site and placed into deep freezers at -20°C before they were transferred within one week to the National Public Health Institute and stored in deep freezers at -70°C. Routine fasting laboratory tests included the concentrations of blood glucose and those of serum total cholesterol and triglycerides (Glucose Hexokinase, Cholesterol CHOD PAP and Triglycerides GPO PAP, Olympus System Reagent, Germany), those of HDL cholesterol and low-density lipoprotein (LDL) cholesterol (HDL-C Plus and LDL-C Plus, Roche Diagnostics GmbH, Germany), and those of gamma-glutamyltransferase (GGT) and uric acid (IFCC/ECCLS and URIKAASI PAP, Konelab, Thermo Electron Oy, Finland).

The diagnostic mental health interview was performed at the end of the comprehensive health examination. The computerized version of the CIDI (M-CIDI) was used. The program uses algorithms to meet the Diagnostic and Statistical Manual of Mental Disorders (DSM-IV) criteria and allows the estimation of DSM-IV diagnoses for major disorders [21]. The translation of the M-CIDI into Finnish was made pair wise by psychiatric professionals and revised by others. The official Finnish translation of the DSM-IV classification was used as a basis for formulating the interview. The process included consensus meetings, third expert opinions, an authorized translator's review, and testing with both informed test subjects and unselected real subjects [22]. Interviews were performed to determine the 12-month prevalence rates of major depressive episodes and disorder, dysthymia, general anxiety disorder, panic disorder with or without agoraphobia, social phobia, alcohol abuse and dependence, and other substance dependence and abuse.

As part of the assessment, the participants filled in the items of lifetime seasonal variations in mood and behavior taken and adapted from the Seasonal Pattern Assessment Questionnaire (SPAQ) [23]. The questionnaire was translated into Finnish and then back-translated to revise the linguistic accuracy. Each of the six items of sleep length, social activity, mood, weight, appetite, and energy level was scored from 0 to 3 (none, slight, moderate or marked change), not from 0 to 4 (none, slight, moderate, marked or extremely marked change), with the sum or global seasonality score (GSS) ranging from 0 to 18. A dichotomous variable depicting seasonality was derived from the distribution of global scores on the modified questionnaire and based on the provisional criteria similar to the original ones [24], the GSS ranging from 0–7 (not affected) and 8–18 points (affected).

There are several definitions for metabolic syndrome and its risk factors. In this study we used US Adult Treatment Panel III of the National Cholesterol Education Program (NCEP-ATPIII) criteria [NCEP 2002] and the International Diabetes Federations (IDF) criteria [IDF 2005] to determine metabolic syndrome.

The US Adult Treatment Panel III of the National Cholesterol Education Program (NCEP-ATPIII) criteria for metabolic syndrome is [NCEP 2002] defined as having at least three of the following components: the fasting blood glucose level 6.1 mmol/l or higher, the high blood pressure (systolic pressure 130 mmHg or more or diastolic pressure 85 mmHg or more), the serum triglycerides level 1.7 mmol/l or higher, the serum high-density lipoprotein cholesterol level lower than 1.0 mmol/l for men or lower than 1.3 mmol/l for women, or the waistline 102.1 cm or more for men or 88.1 cm or more for women.

The International Diabetes Federations (IDF) criteria for metabolic syndrome [IDF 2005] is defined as having waistline of 94 cm or more for men or 80 cm or more for women and at least two of the following components: the serum triglycerides level 1.7 mmol/l or higher, the serum high-density lipoprotein cholesterol level lower than 1.02 mmol/l for men or lower than 1.29 mmol/l for women, high blood pressure in terms of systolic pressure 130 mmHg or more or diastolic pressure 85 mmHg or more or treatment for previously diagnosed hypertension and raised fasting plasma glucose level 5.6 mmol/l or higher, or previously diagnosed type 2 diabetes.

The individual risk factor variables are listed below. These include the variables forming the criteria's above and in addition supplemental variables, that World Health Organization (WHO) and European Group for the Study of Insulin Resistance (EGIR) consider as risk factors for metabolic syndrome and American Association of Clinical Endocrinologists (AACE) use to define Insulin Resistance Syndrome.

The blood pressure was defined high when mean value of systolic blood pressure was 140 mmHg or more or diastolic blood pressure was 90 mmHg or more. A variable taking into account high blood pressure and in addition a treatment for previously diagnosed hypertension was created. We also used a variable which defined blood pressure high when mean value of systolic blood pressure was 130 mmHg or more or diastolic blood pressure was 85 mmHg or more. A variable with preceding and hypertension medication was also included in the study.

The serum high-density lipoprotein (HDL) cholesterol level was considered low when it was lower than 1.02 mmol/l for men or lower than 1.29 mmol/l for women. We also used another variable with thresholds of 1.0 mmol/l and 1.3 mmol/l, respectively. The triglyceride levels were considered raised if they were higher than 1.7 mmol/l in both genders. A variable taking into account raised triglyceride levels and also the low HDL cholesterol in terms of 0.9 mmol/l or less in men and 1.0 mmol/l in women was used. A variable with triglycerides termed high when higher than 2 mmol/l or HDL was less than 1.0 mmol/l or person was using lipid medication was also used in this study.

Plasma glucose levels were measured after fasting at least for 4 hours. The first variable considered fasting plasma glucose levels raised if they were 6.1 mmol/l or higher. The second variable was positive if the fasting glucose levels were between 6.1–6.9 mmol/l. The third variable was positive if fasting plasma glucose levels were 5.6 mmol/l or higher, or the individual had previously diagnosed type 2 diabetes.

Waist circumference was measured in centimeters. We also used two additional variables to define the waistline status: In the first variable circumference was considered high when it was 102 cm or more for men or 88 cm or more for women, in the second variable the values were 94 cm or more for men or 80 cm or more for women. The waist/hips circumference ratio was determined high when it was 0.9 or more for men or 0.85 or more for women.

### Study sample

Overall, the 5480 individuals participated in the health status examination and the diagnostic mental health interview, filled in the self-report of seasonal changes in mood and behavior and gave venous blood samples for DNA extraction and were screened with the M-CIDI interview to have no mental illness according to the DSM-IV criteria. Among these individuals, 517 were randomly selected to form the final study sample.

### Gene and SNP selection

A total of 39 single-nucleotide polymorphisms (SNPs) of 19 genes were genotyped (Table 1). Herein, we wanted to focus on the circadian clock and selected genes which were either canonical circadian clock genes (*Arntl, Arntl2, Clock, Cry2, Npas2, Per2 *and *Timeless*) or genes having their influence on pathways related to the circadian clockwork (*Adcyap1, Drd2, Opn4, Npy, Vip, Vipr2, Fdft1*). Since the circadian clockwork and sleep are interactive, specific sleep-related genes were included (*Acads, Ada *and *Glo1*). *Arntl2 *was included in the study because it has significant homology with *Arntl1 *and *Ncoa *because it has significant sequence homology with *Clock *and therefore a possible role in the circadian clock [25,26]. Both candidate SNPs and tag-SNPs were included in this study. Candidate SNPs were selected based on their possible functional potential including variation resulting in amino acid change (i.e. missense, Table 1) and SNPs previously reported to have relevance to seasonal changes in mood and behavior. HapMap tag-SNPs were selected in order to improve the coverage.

**Table 1:** Genotypes and allele frequencies.

Gene	SNP^a^	Mutation Type	Allele 1^b^	Allele 2	*n *1 (freq)^C^	*n *2 (freq)	*n *11 (freq)	*n*12 (freq)	*n *22 (freq)
*Acads*	rs1799958	missense	G	A	768 (0.74)	266 (0.26)	283 (0.55)	202 (0.39)	32 (0.06)

*Ada*	22G>A	missense	G	A	978 (0.95)	56 (0.05)	461 (0.89)	56 (0.11)	0

*Adcyap1*	rs2856966	missense	A	G	850 (0.82)	184 (0.18)	344 (0.67)	162 (0.31)	11 (0.02)

*Arntl*	rs6486120	intronic	G	T	744 (0.72)	290 (0.28)	280 (0.54)	184 (0.36)	53 (0.10)
	
	rs1982350	intronic	G	A	587 (0.57)	447 (0.43)	181 (0.35)	225 (0.44)	111 (0.21)
	
	rs3816360	intronic	C	T	552 (0.53)	482 (0.47)	152 (0.29)	248 (0.48)	117 (0.23)
	
	rs2278749	intronic	C	T	823 (0.80)	211 (0.20)	328 (0.63)	167 (0.32)	22 (0.04)
	
	rs2290035	intronic	A	T	595 (0.58)	439 (0.42)	175 (0.34)	245 (0.47)	97 (0.19)

*Arntl2*	rs7958822	intronic	G	A	560 (0.54)	474 (0.46)	147 (0.28)	266 (0.51)	104 (0.20)
	
	rs4964057	intronic	T	G	601 (0.58)	433 (0.42)	178 (0.34)	245 (0.47)	94 (0.18)
	
	rs1037921	missense	A	G	947 (0.92)	87 (0.08)	433 (0.84)	81 (0.16)	3 (0.01)
	
	rs2306074	intronic	T	C	668 (0.65)	366 (0.35)	213 (0.41)	242 (0.47)	62 (0.12)
	
	rs35878285	mis-sense	A		1034 (1.00)		517(1.00)		

*Clock*	rs2412646	intronic	C	T	760 (0.74)	274 (0.26)	280 (0.54)	200 (0.39)	37 (0.07)
	
	rs11240	intronic	C	G	696 (0.67)	338 (0.33)	227 (0.44)	242 (0.47)	48 (0.09)
	
	rs2412648	intronic	T	G	654 (0.63)	380 (0.37)	210 (0.41)	234 (0.45)	73 (0.14)
	
	rs3805151	intronic	T	C	613 (0.59)	421 (0.41)	183 (0.35)	247 (0.48)	87 (0.17)

*Cry2*	rs2863712	missense	T		1034 (1.00)		517(1.00)		

*Drd2*	rs1800497	missense	G	A	838 (0.81)	196 (0.19)	336 (0.65)	166 (0.32)	15 (0.03)
	
	rs6277	silent	G	A	542 (0.52)	492 (0.48)	141 (0.27)	260 (0.50)	116 (0.22)

*Fdft1*	rs11549147	missense	A	G	944 (0.91)	90 (0.09)	431 (0.83)	82 (0.16)	4 (0.01)

*Glo1*	rs2736654	missense	T	G	662 (0.64)	372 (0.36)	207 (0.40)	248 (0.48)	62 (0.12)

*Opn4*	rs1079610	missense	T	C	714 (0.69)	320 (0.31)	246 (0.48)	222 (0.43)	49 (0.09)

*Ncoa3*	rs6094752	missense	C	T	1003 (0.97)	31 (0.03)	486 (0.94)	31 (0.06)	0
	
	rs2230782	missense	G	C	932 (0.9)	102 (0.1)	422 (0.82)	88 (0.17)	7 (0.01)
	
	rs2230783	missense	T		1034 (1.00)		517(1.00)		

*Npas2*	rs11541353	missense	C	T	859 (0.83)	175 (0.17)	358 (0.69)	143 (0.28)	16 (0.03)
	
	rs2305160	missense	G	A	727 (0.7)	307 (0.3)	252 (0.49)	223 (0.43)	42 (0.08)

*Npy*	rs16139	missense	T	C	956 (0.92)	78 (0.08)	444 (0.86)	68 (0.13)	5 (0.01)

*Per2*	rs934945	missense	C	T	917 (0.89)	117 (0.11)	402 (0.78)	113 (0.22)	2 (0.004)
	
	10870	intronic	A	G	854 (0.83)	180 (0.17)	350 (0.68)	154 (0.30)	13 (0.03)
	
	rs2304672	UTR 5'	G	C	865 (0.84)	169 (0.16)	361 (0.70)	143 (0.28)	13 (0.03)
	
	S662G	missense	T		1034 (1.00)		517(1.00)		

*Plcb4*	rs6077510	missense	A	G	552 (0.53)	482 (0.47)	142 (0.27)	268 (0.52)	107 (0.21)

*Timeless*	rs2291739	missense	A	G	624 (0.6)	410 (0.4)	193 (0.37)	238 (0.46)	86 (0.17)
	
	rs2291738	intronic	C	T	546 (0.53)	488 (0.47)	147 (0.28)	252 (0.49)	118 (0.23)

*Vip*	rs3823082	intronic	C	T	854 (0.83)	180 (0.17)	351 (0.68)	152 (0.29)	14 (0.03)
	
	rs688136	UTR 3'	T	C	676 (0.65)	358 (0.35)	221 (0.43)	234 (0.45)	62 (0.12)

*Vipr2*	rs885863	UTR 3'	T	C	518 (0.50)	516 (0.50)	126 (0.24)	266 (0.51)	125 (0.24)

a) dbSNP symbols

b) Alleles extracted from HapMap

c) Total number of alleles in study sample, frequencies in parenthesis.

### Genotype analysis

Genomic DNA was isolated from the whole blood according to standard procedures. SNPs were genotyped with a fluorogenic 5' nuclease assay method (TaqMan™) with pre-designed primer-probe kits (TaqMan^® ^Pre-Designed SNP Genotyping Assays) using the Applied Biosystems 7300 Real Time PCR System (Applied Biosystems, Foster City, California, USA) according to the instructions provided by the manufacturer.

Custom TaqMan^® ^SNP Genotyping Assays were used for three SNPs. The primere sequences were CGCACGAGGGCACCAT and TGGGCCCCGCTAAGC and the reporter sequences ACTTTGGGCTTGTCGAA and ACTTTGGGCTTGTTGAA for ADA 22G>A (Asp8Asn), AAGCCGACTTTGCCTGAGT and ACAAGGAGCCGGGTTCTG and the reporter sequences CTTGGGCATTTTCAT and TTGGGCGTTTTCAT for PER2 10870, and GCTCAGCAGCAGCCTGAA and CGAAACTGCGACTGGTCTGATT and the reporter sequences CTTGCTACAAGTATCTC and TTGCTACAGGTATCTC for FDFT1 rs11549147.

All samples were successfully genotyped, yielding the success rate of 100% for all SNPs, and about 5% of samples were re-genotyped to confirm the genotyping results. The following three SNPs were not in the Hardy-Weinberg equilibrium: ARNTL rs1982350 (P = 0.01), ARNTL rs6486120 (P = 0.009) and PER2 rs934945 (P = 0.05).

### Statistical analysis

Genotype frequencies, allele frequencies and Hardy-Weinberg p-values were calculated with the Pearson exact test. Only those haplotypes occurring with a frequency >0.05 were considered. The linkage disequilibrium (LD) between the SNPs analyzed was estimated. The remaining 35 SNPs were tested using additive model. Coefficients, odds ratios (OR) and their 95% confidence intervals (CI) were calculated. The sex and age were controlled for these analyses. The p-values were corrected to reduce the false positives resulting from multiple testing by using an approximation of Bonferroni-p-values: we selected associations with significant p-values and low false discovery rates (FDR below 0.05) and then corrected the p-values with the number of the genes analyzed (17). Statistical analysis was performed using the R software, version 2.5.0 [27], and the PLINK software, version v1.04 [28].

### Ethics

The study project was coordinated by the National Public Health Institute and implemented in collaboration with social insurance organizations and the Ministry of Social Affairs and Health. It provided a written informed consent to each participant, giving a full description of the protocol before signing it. The procedures were according to the ethical standards of the responsible committee on human experimentation and with the Declaration of Helsinki, its amendments and revision.

## Results

The allele frequencies and genotype distributions of the SNPs are shown in Table 1. The first 100 samples genotyped indicated that in our Finnish study population four SNPs were not polymorphic, including *Arntl2 *rs35878285, *Cry2 *rs2863712, *Ncoa3 *rs2230783 and *Per2 *S662G, so these were excluded from further analysis. Each polymorphic SNP was then analyzed in relation to seasonality and to metabolic syndrome risk factors. The significant results are presented in Table 2.

**Table 2:** Results from one-SNP analysis.

Variable	Gene	SNP	P-value	P-value corrected for multiple testing	Beta-coefficient	95% CI
Fasting blood glucose level (Logarithmic) a	*Per2*	#10870	0.002	0.049	-0.010	-0.016–-0.035

						

Variable	Gene	SNP	P-value	P-value corrected for multiple testing	Odds ratio	95% CI

High blood pressure b	*Npas2*	rs11541353	0.001	0.02	0.54	0.37–0.79

High blood pressure or hypertension medication c	*Npas2*	rs11541353	<0.001	0.015	0.53	0.36–0.77

Single SNPs were analyzed using linear regression for continuous variables and logistic regression for dichotomous variables. Betacoefficients were calculated for continuous variables, odds ratios for dichotomous variables. The sex and age were controlled for these analyses. P-values corrected for multiple testing were calculated.

a) The concentrations of blood glucose (mmol/L) after fasting at least 4 hours and without drinking on the same day. The variable was log-transformed to obtain the normal distribution.

b) The blood pressure was defined high when systolic pressure was 140 mmHg or higher or diastolic pressure was 90 mmHg or higher.

c) High blood pressure (b) or treatment for previously diagnosed hypertension.

We found associations with circadian clock genes and the risk factors for metabolic syndrome. *Npas r*s11541353 was associated with hypertension, the minor allele being protective against hypertension (T vs. C, OR = 0.54, Corrected P-value = 0.02). The results almost the same when people getting treatment for their hypertension were included in group (T vs. C, OR = 0.53, Corrected P-value = 0.015). *Per2 *10870 was associated with glucose metabolism. 10870 minor allele reduced the risk of raised plasma glucose (G vs. A, Beta coefficient = -0.010, Corrected P-value = 0.049).

## Discussion

Our main results herein are that *Npas2 *is linked to hypertension and that *Per2 *is associated with blood glucose levels.

Seasonality and disruption of circadian molecular clockwork are risk factors for metabolic syndrome ([12,20]. We now found that the common risk factors for metabolic syndrome are associated with polymorphisms in circadian clock genes. *Npas2 *rs11541353 was associated with hypertension in the Finnish population. Earlier, *Arntl *was linked to hypertension and type 2 diabetes mellitus [29]. Now, we demonstrate herein the associations of *Npas2 *with hypertension and of *Per2 *with blood glucose levels.

Together these earlier findings and those of ours emphasize the importance of the circadian system and its core genes in regulation of blood pressure, and point to a role in pathological situations. Moreover, they parallel to SAD in which there is a strong metabolic component and with which this unit of ARNTL, NPAS2 and PER2 is associated [30]. There are often not only disturbances in the metabolic networks [31] but also disruptions of the circadian rhythms [32] together with pronounced seasonal changes in mood and behavior [33] in individuals having affective disorders. Now, this may concern the general population as well.

Npas2 rs11541353 is a missense mutation, leading to substitution of serine with leusine in the amino acid position 471. *Npas2 *rs11541353 minor allele was protective against hypertension and heterozygosity of Npas2 rs11541353 is protective against Seasonal affective disorder (SAD) [30]. These findings reveal that protection from seasonal variations and protection from high blood pressure go hand in hand in some cases. However, Partonen *et al*. also found that homozygosity for both *Npas2 *rs11541353 minor and major alleles was a major risk factor for SAD. Combining these results, persons with two major alleles of *Npas2 *rs11541353 have substantially increased risk not only for SAD but also for hypertension. However, when a person has two *Npas2 *rs11541353 minor alleles, the results are difficult to interpret, as the homozygosity increases the odds for SAD, but protects against hypertension. Next, the phenotypes in terms of SAD and hypertension in *Npas2 *rs11541353 homozygous and heterozygous persons need to be analyzed.

Our results indicate, that *Per2 *10870 contributed to changes in glucose metabolism. *Per2 *10870 is an intronic mutation originally found by Spanagel *et al *(2005), when searching for the *Per2 *SNPs modulating alcohol intake in mice. Its minor allele G was protective against high alcohol intake in humans [34] but increased the odds for SAD [30]. In our current study, the minor allele G reduced the risk for raised plasma glucose levels. Lamia *et a*l. previously demonstrated that Per1-/-;Per2-/- mice have altered blood glucose homeostasis [35]. Another recent study demonstrated that administration of metformin, one of the most commonly used drugs for type 2 diabetes, leads to the degradation of PER2 and to a phase advance in the circadian gene expression [36]. It remains to be elucidated whether PER proteins are independently important for glucose homeostasis or does their role in the circadian clock lead to the effects seen.

Woon *et al*. found association between *Arntl *and hypertension and type 2 diabetes mellitus [29]. Our SNP selection did not include the SNPs used in their study, which can explain why we failed to see any associations. Recent studies have also found association between *Clock*-gene polymorphism and the metabolic syndrome in man [13,14]. It is of note that we did not find support to these links in our study. We did, however, find several interesting associations, which failed to show statistically significant p-values after correction (Table 3). These include associations between *DRD2 *rs6277 and blood glucose levels, *PLCB4 *rs6077510 and *Per2 *SNP rs934945 and waist circumference, and *Vipr2 *rs885863 and low HDL cholesterol level. In addition, *Per2 *SNP rs934945 was associated with the metabolic syndrome.

**Table 3:** Single SNP analysis with corrected p- values = 0.10.

Variable	Gene	SNP	P-value	P-value corrected for multiple testing	Beta- coefficient	95% CI
Fasting blood glucose level (Logarithmic) a	*DRD2*	rs6277	0.003	0.051	-0.008	-0.012–-0.003

Waist circumference b	*PLCB4*	rs6077510	0.004	0.085	2.0	0.63–3.4

						

Variable	Gene	SNP	P-value	P-value corrected for multiple testing	Odds ratio	95% CI

High waist circumference c	*Per2*	rs934945	0.003	0.062	1.9	1.2–3.0

Low HDL cholesterol d	*Vipr2*	rs885863	0.004	0.069	1.5	1.1–2.0

Metabolic syndrome [IDF] f	*Per2*	rs934945	0.004	0.070	1.9	1.2–2.9

Single SNPs were analyzed using linear regression for continuous variables and logistic regression for dichotomous variables. Betacoefficients were calculated for continuous variables, odds ratios for dichotomous variables. The sex and age were controlled for these analyses. P-values corrected for multiple testing were calculated.

a) The concentrations of blood glucose (mmol/L) after fasting at least 4 hours and without drinking on the same day. The variable was log-transformed to obtain the normal distribution.

b) Waist circumference in centimeters

c) Waist circumference 94 cm or more for men or 80 cm or more for women

d) Serum high-density lipoprotein (HDL) cholesterol level was considered low when it was lower than 1.02 mmol/l for men or lower than 1.29 mmol/l for women.

f) Metabolic syndrome was assessed using the International Diabetes Federations (IDF) criteria [IDF 2005] and defined as having waistline of 94 cm or more for men or 80 cm or more for women and at least two of the following components: the serum triglycerides level 1.7 mmol/l or higher, the serum high-density lipoprotein cholesterol level lower than 1.02 mmol/l for men or lower than 1.29 mmol/l for women, high blood pressure in terms of systolic pressure 130 mmHg or more or diastolic pressure 85 mmHg or more or treatment for previously diagnosed hypertension and raised fasting plasma glucose level 5.6 mmol/l or higher, or previously diagnosed type 2 diabetes.

There are some limitations in our study. We relied on a self-report questionnaire when assessing the seasonal variations in mood and behavior. However, this questionnaire has been reported to have high sensitivity and specificity [37] and can be regarded as valid for the lifetime-retrospective assessment of routine seasonal variations in mood and behavior.

Our study bears several strengths. This was a population-based and nation-wide study. Its sample size was relatively big and representative of the general population aged over 30 living in a northern European country, Finland. Hence, these data can be generalized directly to concern the whole adult population of Finland, or any population having similar living conditions. We had rich phenotype data with reliable laboratory tests and valid assessments of syndromes on our focus. The single-nucleotide polymorphisms used were selected for their potential role in the function of the gene, which augments the possibility that the genotype seen here contributes to the phenotype although experimental analysis is needed for verification.

## Conclusion

Our findings herein link the circadian gene variants and risk factors of the metabolic syndrome. *Npas2 *was associated with hypertension and *Per2 *with blood glucose levels. Our findings give support to the view that there are relevant relationships between circadian clocks and metabolic syndrome.

## Competing interests

JH has served as consultant to Janssen-Cilag, other authors have no conflicts of interests.

## Authors' contributions

AE drafted the manuscript. LK carried out the genotyping and helped to draft the manuscript. AE, LK, JH and TP participated in the design of the study and performed the statistical analysis. TP, STS, JL, AR and AA conceived of the study, and participated in its design and coordination and helped to draft the manuscript. All authors read and approved the final manuscript.
